# Recycling of Bottle Grade PET: Influence of HDPE Contamination on the Microstructure and Mechanical Performance of 3D Printed Parts

**DOI:** 10.3390/polym14245507

**Published:** 2022-12-15

**Authors:** Joanne Vaucher, Adrien Demongeot, Véronique Michaud, Yves Leterrier

**Affiliations:** Laboratory for Processing of Advanced Composites (LPAC), École Polytechnique Fédérale de Lausanne (EPFL), CH-1015 Lausanne, Switzerland

**Keywords:** recycling, contamination, poly(ethylene terephthalate), high density polyethylene, 3D printing

## Abstract

As part of a project that aims to provide people with disabilities with simple assistive devices in Colombia, the possibility of creating a PET filament that can be printed by Fused Deposition Modelling (FDM) from beverage bottle waste was investigated, with the aim to remain as simple as possible in terms of plastic collection, sorting, processing, and printing. Recycled PET filaments were thus produced by extrusion from collected PET bottles, with the potential addition of HDPE, which comes from caps and rings. The microstructure, mechanical performance, and printing quality of parts produced with these filaments were investigated in comparison to commercial PET virgin and recycled filaments. HDPE presence as an immiscible blend did not affect the ease of extrusion or the quality of the printing, which were all satisfactory. In some conditions, the addition of 5 wt% of HDPE to recycled PET had a toughening effect on otherwise brittle samples. This behavior was attributed to the presence of elongated HDPE inclusions resulting from shear forces induced by the layer-by-layer printing, provided that the interface temperature remained high between layer depositions. This confirms that the mechanical performance of recycled PET is very sensitive to the processing conditions, especially in the case of 3D printing. Nonetheless, this low-cost process that did not require sophisticated compatibilization schemes allowed for the printing of parts with mechanical properties comparable to those obtained with high purity, commercially recycled filaments, opening interesting perspectives for a low-cost PET recycling process.

## 1. Introduction

In the last decade, the production of polymer-based materials has significantly increased. In 2019, the global plastic production reached 370 million tons, and this amount is expected to increase to 900 million tons by 2050 [1,2]. Packaging represents the dominant application market, accounting for more than 40% of the global consumption of plastics [3]. Polyethylene terephthalate (PET) is one of the most favorable packaging materials, thanks to its light weight, clarity, and barrier properties against moisture and oxygen [4]. PET packaging represents almost half of single-serve beverage packaging and 12% of global solid waste [5]. The consequences of the worldwide use of plastic on the environment are important, especially in landfills and oceans, and the urge to develop efficient recycling solutions is central to the scientific community. Recycling of PET is already a widely developed field worldwide, and various routes, including mechanical, chemical, and biocatalytic recycling, are investigated [6,7]. In Switzerland, more than 80% of the consumed PET bottles are collected and recycled, mainly into new beverage bottles [8,9]. This closed-loop recycling process was optimized in terms of material purity to avoid contamination issues with foreign materials and molecular weight control to guarantee stable processing and performance. Indeed, contamination of PET with other types of polymers, such as high-density polyethylene (HDPE), coming, for instance, from the bottles’ caps, affects the mechanical properties of injection-molded recycled PET due to phase separation in the immiscible blend [10]. The compatibilization of these two polymers using reactive modifiers such as maleic anhydride is well-established but requires accurate processes [11,12,13].

A novel method for processing virgin and recycled PET is fused deposition modeling (FDM) [14,15], one of the leading additive manufacturing processes in which layers of an extruded filament of the polymer are applied sequentially on a carrier into a predefined pattern, with the potential to create any 3D architecture. The use of PET in 3D printing is not widespread nowadays, primarily because of its semi-crystalline nature, and the influence of contamination on the quality of printed parts has not been explored. Indeed, semi-crystalline polymers often negatively impact the printing process, for instance in cases of excessive shrinkage [16] and may result in brittle parts. In fact, polyethylene terephthalate glycol-modified (PETG) is usually preferred in 3D printing, as the substitution of ethylene glycol by cyclohexanedimethanol in the PET backbone makes it more flexible and less brittle when printed [17,18,19].

As part of a project that aims to provide people with disabilities with simple assistive devices in Colombia, the possibility of creating a printable PET filament from beverage bottle waste was investigated, since the availability of commercial filaments is limited in many remote areas. The novelty was to check whether the contamination of the recycled PET with HDPE would be critical for the mechanical performance of printed parts in the absence of compatibilization. Indeed, the process had to be as simple as possible due to the limited infrastructure available, both in terms of plastic collection, sorting, processing, and printing. Thus, this study focuses on the low-cost recycling of bottle-grade PET into filaments for 3D printing by FDM, with a focus on the effect of controlled concentrations of HDPE on the structure and properties of 3D printed parts. The interplay between printing conditions, blend microstructure, and resulting mechanical properties is analyzed to propose optimal compositions and printing conditions. This investigation shows that it is possible to 3D-print HDPE-contaminated PET parts with excellent mechanical performance, but only under specific process conditions.

## 2. Materials and Methods

### 2.1. Materials and Processes

PET and HDPE were collected from used 500 mL bottles at the EPFL in Lausanne, Switzerland. All labels were torn off by hand, and surfaces with remaining traces of glue were cut out. The HDPE cap and ring closure were carefully separated from the PET bottles, and both types of plastic were washed with cold water and soap, then dried in the air at room temperature for at least 24 h. The two polymers were ground using a shredder (150 Series, Rapid Granulator, Sweden) with a 5 mm-diameter hole perforated screen. No additional drying was performed on the materials prior to subsequent processing and testing. This was a deliberate choice due to the limited infrastructure available for disabled people in Columbia, which could impact the final performance of the printed materials [20]. The ground polymer flakes were mixed with a spoon in batches of 50 g to achieve controlled concentrations of HDPE in PET ranging from 0 to 10 wt%. The polymer mixtures were extruded into filaments with a compounding twin screw extruder (Prism TSE 16 TC, Thermo Fisher Scientific) at temperatures of 225 °C at the feeder, 230 °C in the center, and 235 °C at the die, with a screw velocity of 30 rpm. Cooling of the filaments was carried out on a rolling bed at ambient temperature with the help of two domestic fans. The diameter of the die was 2 mm, and the targeted filament diameter was 1.75 mm. A summary of the various types of filaments is given in Table 1. In order to facilitate the identification of the contamination level, HDPE batches were sorted by color, and each blend was prepared with a different color. In preliminary tests, it was found that the effect of the color pigment in the HDPE phase was negligible, as could be expected due to the immiscible nature of HDPE, combined with its rather low concentration. For comparison purposes, commercial filaments of virgin PET (EPR InnoPET, Innofil) and recycled PET (rPET Natural Blue) were purchased from BASF 3D Printing Solutions, The Netherlands.

Samples were printed from commercial and home-extruded filaments with a Prusa i3 MK3 printer (Czech Republic) at die and bed temperatures of 265 °C and 100 °C, respectively. A 0.4 mm diameter die and a standard polyetherimide bed, wiped with a commercial cleaning agent, were used. A homemade insulating box with a metallic frame on which 40 mm-thick extruded polystyrene boards were assembled to cover each face was placed around the printer in order to control the surrounding temperature. The printing quality was investigated through the analysis of 3D benchy models. Those objects representing a tugboat were designed on purpose to test the printer’s accuracy and contained most of the difficult-to-print features, such as symmetry, overhanging curved surfaces, smooth surfaces, holes, etc. [21]. Each 3D benchy model was printed with a layer height of 0.2 mm, 10% infill, an extrusion print speed of 50 mm/s, and a travel speed of 120 mm/s. Specimens for tensile testing were printed in the 0° direction (parallel to tensile testing) with an infill of 100% and a randomized infill start for all types of filaments. Measurements were carried out on 3 different specimens for each batch. Two series were prepared: in the first one, specimens were printed one by one, with 27 s of cooling time between the deposition of consecutive layers, while in the second one, 3 specimens were printed simultaneously each time, with 85 s of cooling time between consecutive layers. These two series are respectively termed ‘single prints’ and ‘3 prints’ in the following.

Injection-molded specimens were also prepared with cuts of E-PET0 and E-PET5 filaments using a Micro Injection Moulders Xplore IM 5.5 (the Netherlands), with a melt temperature of 270 °C, a mold temperature of 80 °C, and a pressure of 8 bar maintained for 7 s.

### 2.2. Characterization Methods

The polymer samples were characterized using different methods, as summarized in Table 2. Thermogravimetric analysis (TGA) of polymer flakes was performed on a Perking Elmer TGA 4000 with a sensitivity of 1 µg. The samples were heated from 30 to 600 °C at a rate of 10 °C/min under a 20 mL/min air flow to mimic the extrusion environment. A second analysis was performed according to the following heating procedure: heat from 30 to 265 °C with a rate of 10 °C/min, then hold at this temperature for 30 min. This temperature corresponds to the printing temperature of the filament, and the experimentation was done in order to highlight any degradation that would not occur instantaneously. The samples were not dried before the measurements in order to replicate the real processing conditions.

Tensile tests were performed according to the ISO 527-1 standard [22] on type 5A specimens (gauge length and width of 25 mm and 6 mm, respectively) with a ZWICK 10 kN uniaxial testing machine (Germany), using clip-on extensometers. Measurements were carried out on 3 different specimens for each batch, along the 0° printing direction. Tensile strength, elongation at break, and Young’s modulus were determined as recommended by the ISO 527-1 standard [22].

Differential scanning calorimetry (DSC) measurements were performed on 10 mg samples using a DSC Q100 from TA Instruments (USA) following a first heating cycle from 25 to 300 °C at 10 °C/min, a cooling to 25 °C at the same rate, and a second heating equal to the first one. One sample was tested for each composition and processing condition. The glass transition temperature (Tg) of PET and the melting point (Tm) of both HDPE and PET were determined during the first heating step, while the crystallization temperature of PET (Tc) was determined during the cooling step. The degree of crystallinity of PET (Xc) was calculated from the first heating cycle as expressed in Equation (1) [23]:

(1) $$X_{c}\left(\%\right)=\frac{\Delta H_{f}-\Delta H_{cc}}{\Delta H_{f}^{0}}\times 100\%$$

where $\Delta H_{f}$ is the enthalpy of fusion, $\Delta H_{cc}$ is the enthalpy of cold crystallization, and $\Delta H_{f}^{0}$ is the reference heat of melting of 100% crystalline PET (140.1 J/g [24]). The degree of crystallinity of PET was also calculated during cooling and the second heating without any cold crystallization component.

Scanning electron microscopy (SEM) observations of fractured surfaces were performed with a Gemini-SEM 300 from Zeiss (Germany). The various specimens were dipped in liquid nitrogen for about 15 min, then fractured in order to obtain observable surfaces. A 20 nm gold layer was coated on all samples with a Quorum multi-coater. Observations were carried out on filaments, printed, and injected specimens.

## 3. Results and Discussion

### 3.1. Thermal Stability

**Figure 1:**
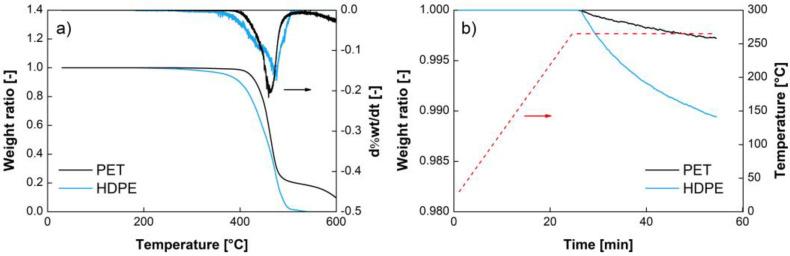
TGA and its derivatives of extruded PET and HDPE at 10 °C/min (**a**) and at 265 °C (**b**).

The TGA data are shown in Figure 1. Figure 1a reveals the absence of significant amounts of water in the polymers, with no detectable evaporation around 100 °C in spite of the fact that the samples were not dried. The equilibrium water concentrations in HDPE and PET (with a crystallinity of 52%) at 23 °C and 50%RH are equal to 6 10^−5^ g/g [25] and 10^−3^ g/g [26], respectively. These amounts are below the detection limit of the TGA (HDPE) and barely detectable (PET). The figure also confirms that no significant degradation of the recycled polymers occurred below 300 °C. Moreover, Figure 1b shows that the selected processing temperatures remained adapted for contaminated blends, as HDPE did not degrade at 265 °C, even after 30 min.

### 3.2. Quality of Extrusion and Printing

**Figure 2:**
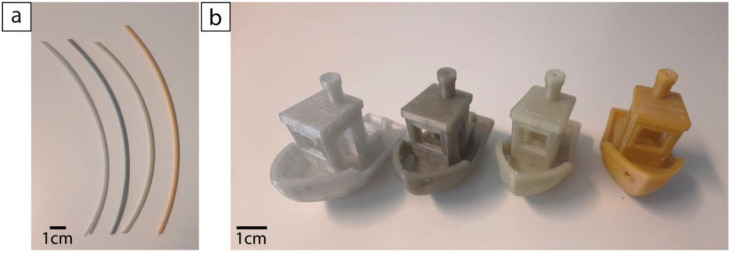
Filaments E-PET0, E-PET2, E-PET5, and E-PET10, from left to right (**a**), and 3D benchy models printed with recycled PET contaminated with 0%, 2%, 5%, and 10% HDPE, from left to right (**b**).

All types of filaments were extruded and printed with the same process parameters. Interestingly, it was observed that the blends with the highest levels of HDPE contamination (5 and 10%) were easier to handle compared with filaments with lower amounts or without HDPE. Indeed, the latter filaments often broke during winding, making it more difficult to have a continuous and easy to roll filament. Nonetheless, all filaments, regardless of the amount of HDPE, could be used in the printer and produce high-quality prints. The average diameter and standard deviation for extruded filaments with different levels of HDPE contamination are reported in Table 1, while the filaments are shown in Figure 2. The 3D benchy models printed with these four types of filaments are also shown in Figure 2. All came out without any trouble, with a quality that was not affected by the contamination. From a total of 13 dimensions, which were measured, the average standard deviation between the measured and expected values was 1.6% for both the pure recycled PET and the blend containing 2% of HDPE, 2.5% for the 5% HDPE blend, and 2.2% for the one with 10% HDPE. Those results were in the same range as the ones obtained for commercial virgin (1.9%) and recycled (2.2%) filaments.

### 3.3. Mechanical Properties

**Figure 3:**
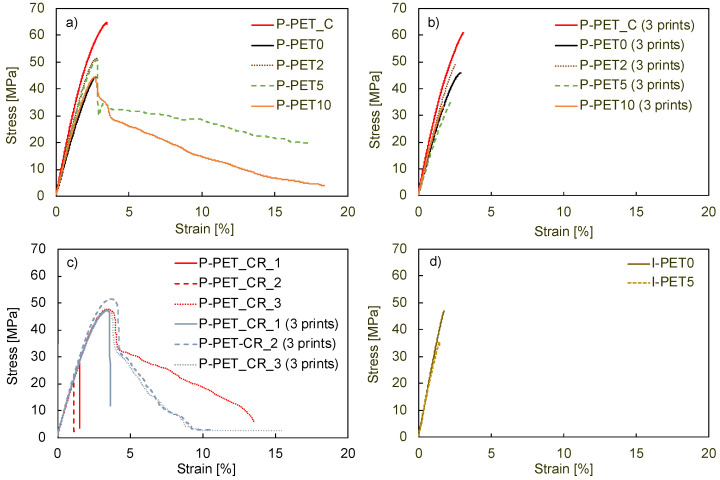
Stress-strain curves of (**a**) single prints, (**b**) three prints, (**c**) prints of commercial recycled filament (single prints and three prints), and (**d**) injected specimens.

The tensile behavior of all printed and injection-molded specimens is shown in Figure 3, and their Young’s modulus and maximum stress are reported in Table 3. The brittle behavior of P-PET0 is evident, with a strain at failure of 2.5%. The addition of 2 wt% of HDPE led to a slight increase in the Young’s modulus, but the samples remained brittle. In contrast, additions of 5 wt% and 10 wt% led to a spectacular increase in the strain at failure, well beyond the yield point. Such results were unexpected and contradict the ductile-to-brittle transition systematically observed with increasing HDPE fraction in the case of immiscible HDPE-PET blends [12,13,27]. These samples exhibited a large drop in the stress at 2.5% strain, which then gradually decreased. The reason for this unexpected toughening mechanism in the case of immiscible blends is further explored in the following.

The Young’s modulus of the single-print blends was equal to 2.1 GPa for all HDPE concentrations, higher than that of pure PET, which was equal to 1.7 GPa. The maximum stress in these materials also increased with HDPE concentrations up to 5 wt%, from about 45 MPa for P-PET0 to about 50 MPa. These values were lower than those of specimens printed with the commercial, virgin PET filament and comparable to the values obtained with the commercial, recycled PET filament. However, these commercial filaments systematically led to brittle specimens, and only filaments containing HDPE led to more ductile, single-printed samples.

Nevertheless, it turned out that printing artefacts had a major impact. Indeed, Figure 3b shows that the three-print samples (i.e., three specimens printed simultaneously rather than one by one) were all brittle, regardless of the amount of HDPE used. Moreover, the maximal stress reached for P-PET5 and P-PET10 was much lower than for P-PET0. The number of simultaneously printed specimens was the only parameter that changed compared to the samples analyzed previously. A possible explanation for the lack of ductility in these samples is that the printed layers cooled down to a lower temperature when multiple specimens were printed simultaneously. Indeed, printing a larger surface at the same speed took more time, which means that the successive layers were applied to a cooler surface. This difference in temperature presumably weakened the interface between two printed layers, inducing a brittle fracture, as previously analyzed by Bakir et al. [28]. This observation highlights the importance of adapted printing parameters and the considerable sensitivity of 3D printing with recycled and contaminated materials. However, the number of specimens that were simultaneously printed did not affect the properties of samples printed with the commercial virgin PET filament.

Figure 3c shows the stress-strain curves of all single-print and three-print specimens based on the commercial recycled filament. The first observation is the low repeatability of the results. Indeed, two of the three single-print samples were brittle, as was one of the three-print samples. The other three samples showed a more ductile behavior and up to 10% plastic deformation. The maximum stress reached, however, was much lower than for virgin PET, with an average drop of about 15 MPa. The elastic modulus also significantly decreased compared to virgin PET, dropping from 2.7 to about 2 GPa for both single-print and three-print specimens. Even though even higher elongations at break were reached in other studies [28], the values obtained here with a commercial recycled filament are comparable to the behavior observed for homemade, contaminated recycled PET filaments. Figure 3d shows that the injection-molded samples of pure recycled PET and recycled PET with 5 wt% HDPE contamination were also brittle. The tensile strength of the pure PET samples was 45 MPa, which corresponded to the value of similar materials [29]. The addition of 5 wt% of HDPE led to a large reduction in tensile strength, in contrast to the single-print samples.

To summarize, these results indicate that contamination with HDPE may lead to a considerable and unexpected brittle-to-ductile transition in the otherwise brittle PET, but only under specific printing conditions. The contamination reduced the strength of injection-molded samples, leading to properties that are similar to those of printed recycled PET in the 0° direction with 100% infill.

### 3.4. Microstructural Analyses

**Figure 4:**
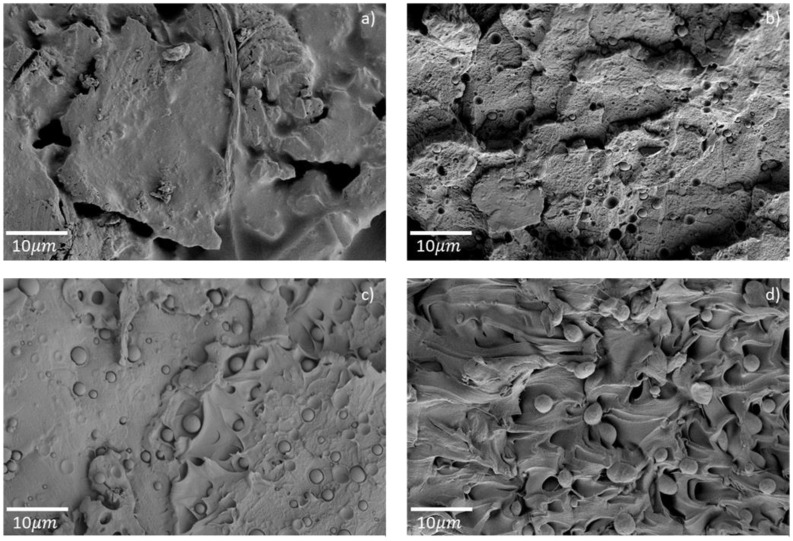
Electron micrographs of extruded filaments for (**a**) E-PET0, (**b**) E-PET2, (**c**) E-PET5, and (**d**) E-PET10.

HDPE is immiscible with PET, as clearly observed on the micrographs of extruded filaments in Figure 4. There are spherical HDPE inclusions with diameters ranging from a few hundred nanometers to two micrometers that are not bonded to the PET. Inclusions smaller than 1 µm were present in the filaments E-PET2 and E-PET5, whereas inclusions in E-PET10 were all larger than 3 µm. These morphologies are characteristic of immiscible polymer blends, as reported in numerous works [12,13,30,31].

**Figure 5:**
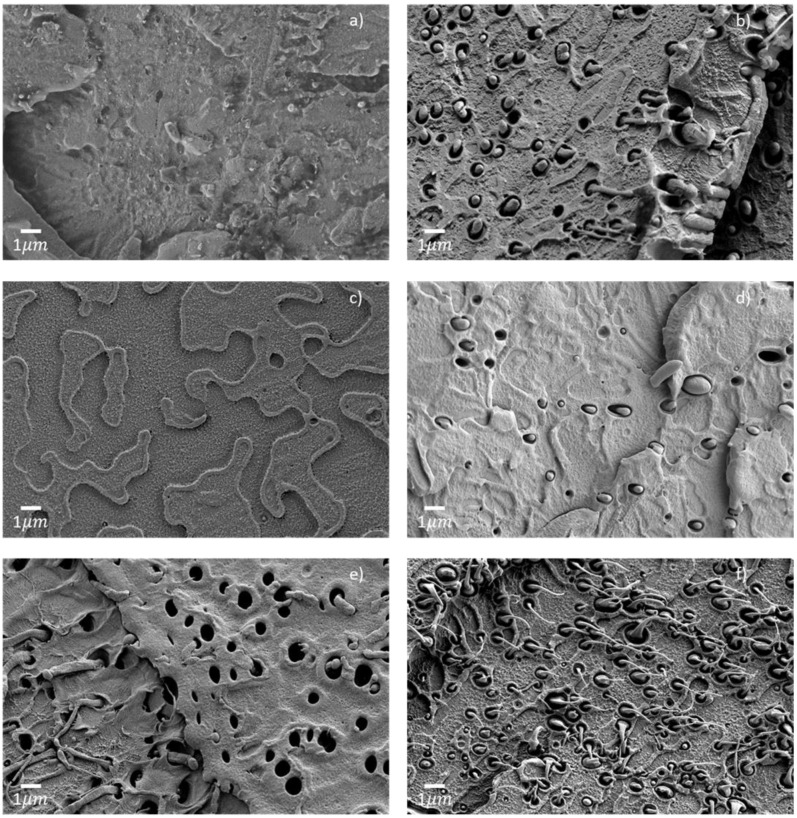
Electron micrographs of single prints (**a**) P-PET0, (**b**) P-PET5 and three prints, (**c**) P-PET0, (**d**) P-PET2, (**e**) P-PET5, and (**f**) P-PET10.

**Figure 6:**
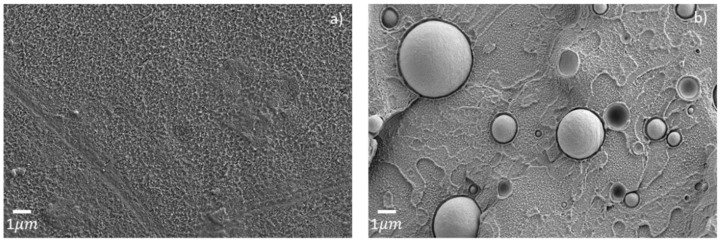
Electron micrographs of injected specimens (**a**) I-PET0 and (**b**) I-PET5.

Quite remarkably, the HDPE inclusions were elongated and thinner in all printed specimens, as shown in Figure 5. This unique morphology is likely to result from the shear forces induced during printing within a die smaller than the filament diameter, causing the observed deformation of the HDPE inclusions [13]. The same tendency was observed for both single-print and three-print batches, with similar elongated inclusions for both P-PET5 specimens (Figure 5b,d). Moreover, the size of the inclusions, around a few hundreds of nanometers, did not seem to depend on the contamination level, whereas their number increased accordingly. This is because the droplet size mostly depends on shear rate, interfacial tension, and the viscosity of the two phases and not on concentration [30]. Aside from this, a gap was observed between the PET and HDPE phases for all types of printed specimens. This could be attributed to a difference in crystallization shrinkage and thermal contraction between these two polymers during the fast cooling, as this phenomenon was not observed in the filament or the injected specimens, which had a slower cooling rate. In the latter case, large HDPE inclusions in the range 1–4 µm were observed in fewer numbers than in printed specimens, as seen in Figure 6b for the sample contaminated with 5 wt% of HDPE, presumably owing to different shear rates between the two types of processes [30]. The morphology of the PET phase shown in Figure 6 was comparable between the pure specimen (I-PET0) and the contaminated one (I-PET5).

The peculiar microstructures could explain the mechanical behavior of contaminated recycled PET. The large HDPE inclusions found in the injected specimen weakened the material [12,13,31]. In printed specimens, small inclusions found in small amounts, like in the 2 wt% HDPE contaminated sample, weakened the material as well. However, when the number of these inclusions increased (P-PET5 and P-PET10), HDPE acted locally as a plasticizer and allowed the material to undergo plastic deformation. As the microstructure did not change between single-print and three-print samples, the brittleness observed in the latter case could be attributed to poor interlayer adhesion.

### 3.5. Thermal Transitions and Crystallinity

**Figure 7:**
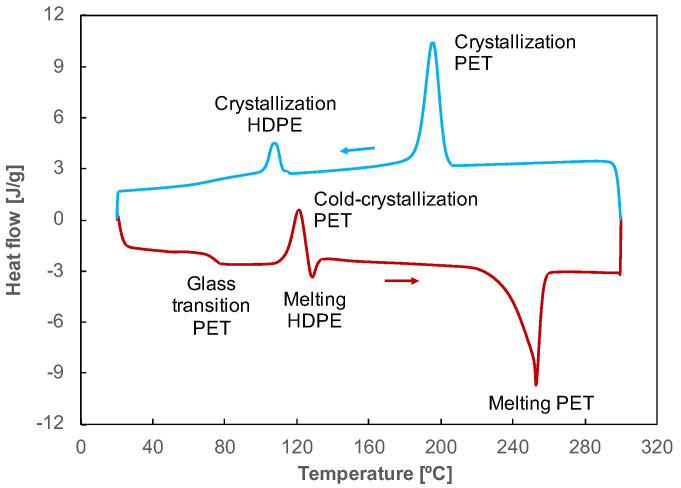
Heating (red curve, down) and cooling (blue curve, up) thermograms of a part printed with a filament of PET contaminated with 5 wt% HDPE.

The crystallinity in the various blends was investigated using DSC to better understand the interplay between the observed morphologies and the brittle-to-ductile transition. Figure 7 shows the thermogram of a part printed with a filament of PET contaminated with 5 wt% HDPE (P-PET5), where the main transitions of the two phases are labeled.

**Figure 8:**
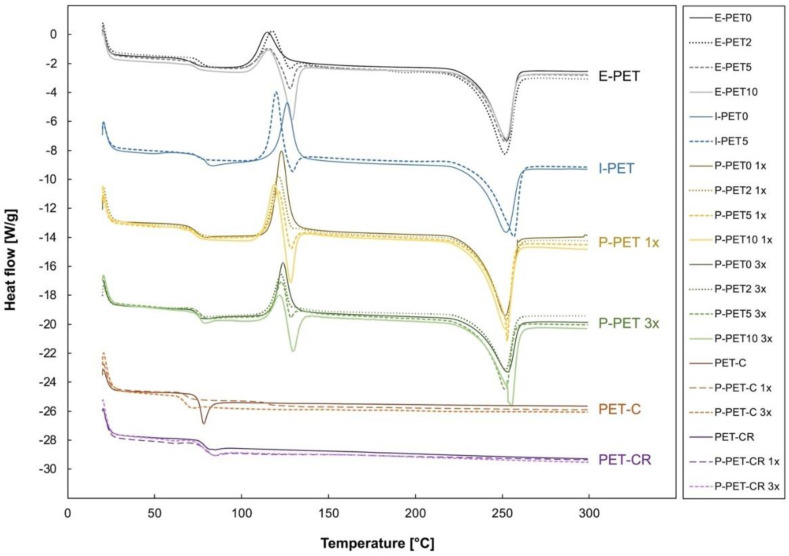
First heating thermograms of extruded, printed (single prints 1× and three prints 3×), and injection molded PET filaments and samples with different levels of HDPE contamination and of virgin and recycled commercial PET, both extruded and printed.

The first heating thermograms of all investigated materials are reproduced in Figure 8, and corresponding thermal transitions and PET crystallinity are summarized in Table 4. The degree of crystallinity of the pure, non-contaminated PET was quite different between the different processes, with the single-print samples with rapid cooling (P-PET0 1×) being much lower (13%) than the other three types of samples, which experienced slower cooling (around 20%).

Regarding the contaminated specimens, the fact that the melting point of HDPE is close to the cold crystallization of PET makes the interpretation of the thermograms more challenging, especially when the amount of HDPE becomes significant (above 5%). Indeed, both transitions occurred around 120 °C, which must be considered when interpreting the values reported in Table 4. In general, the degree of crystallinity appears to increase with increasing HDPE concentration in both filaments and printed parts. Indeed, HDPE inclusions may have acted like germination points for crystallization. Only the results for the injected parts were unexpected, as the degree of crystallinity of I-PET5 was one of the lowest observed in this study, namely 16%. The crystallinity of the PET itself (calculated from the cooling curve) was constant at around 26%, no matter the process or the amount of contamination. This value is comparable to recently reported crystallinities on filaments made from extruded PET bottles [14,32]. Only injected specimens displayed a larger degree of crystallinity (around 35%) for both I-PET0 and I-PET5.

In summary, neither the process nor the HDPE contamination significantly affected the glass and melting temperatures of the recycled PET. Knowing this and considering that the amount of HDPE was not high enough to influence the melting point of PET, the printing and injection temperatures did not have to be adapted when HDPE was added to the recycled PET blends.

A correlation was observed between crystallinity and the mechanical properties, as reported in previous investigations [14,33,34,35]. Indeed, the printed samples that were able to undergo plastic deformation (P-PET5 and P-PET10 single prints) were those with the highest degree of crystallinity. However, this tendency was not observed for the three prints and the injected samples, which were brittle regardless of crystallinity and contamination. The fact that no significant differences in the degree of crystallinity were observed between single-print and three-print samples indicates that their brittleness was not only related to crystallinity. Even though layers were printed on a cooler surface when three samples were printed simultaneously, the degree of crystallinity of recycled PET contaminated with HDPE did not change. Indeed, there were 27 s of delay between the printing of two consecutive layers with single printing, which corresponds to the time for the filament to cool down from the die temperature (265 °C) to the bed temperature (100 °C) [36,37,38]. This interval was more than three times longer when three samples were printed simultaneously. The interface temperature between adjacent layers at the time of printing was therefore higher for single prints, which favored interlayer adhesion and reduced the interlayer stress [39,40,41]. If poor interlayer adhesion was the reason for the brittleness of some printed recycled PET samples, crystallinity was not involved, as confirmed by the performance of the commercial PET samples, which were not crystalline.

Indeed, as shown in Figure 8, not only the commercial virgin and recycled filaments but also the parts printed with these two materials were completely amorphous. This is a major difference between homemade and commercial filaments and prints. As all printed specimens were processed with the exact same parameters, it suggests that the crystallinity of the commercial filaments was related to their composition rather than to processing conditions (e.g., melting temperature or cooling rate). The only consequence of processing seemed to be the lowering of the glass transition temperature of the virgin PET filament when it was printed (going from 76.1 to 68.4 °C), as shown in Table 4. As mentioned in the introduction, one of the main challenges of using PET as a feedstock for 3D printing is its crystallinity, so it is not surprising that commercial filaments are developed in a way that avoids crystallization. Nevertheless, the fact that similar-quality devices could be obtained with recycled PET contaminated with significant amounts of HDPE, regardless of its crystallinity, was a promising observation for further developments.

## 4. Conclusions

The present investigation of the influence of HDPE contamination and process parameters on the microstructure, mechanical performance, and printing quality of 3D printed parts made of recycled bottle-grade PET leads to the following conclusions.

The contamination with HDPE, which came mainly from rings and caps, which are available in the same waste stream, did not affect the ease of extrusion or the printing quality. At contamination levels below 2 wt%, the brittleness of the printed samples increased, with a strain at failure of 2.5%, but these were highly ductile at higher contamination levels, with a strain at failure above 15%. Moreover, the elastic modulus of PET with 5 wt% of HDPE was higher than that of pure recycled PET. Such considerable toughening behavior was attributed to the presence of elongated HDPE inclusions in the immiscible blend resulting from shear forces induced by the printing, and to differences in the PET crystalline structure. This was in fact true only for specific temperature conditions during the printing of successive layers. In the case of three parts printed in parallel with an 85 s cooling time between adjacent layers, the lower temperature of the interface resulted in weaker interfaces and brittle parts. In contrast, when single parts were printed sequentially with a cooling time of 27 s, high enough interfacial temperatures could be maintained throughout the printing cycle.

This investigation confirmed that the mechanical performance of recycled PET was very sensitive to the processing conditions, especially in the case of 3D printing. However, a remarkable toughening effect was uncovered in the contaminated blend, in spite of its immiscible character, due to a process-induced microscale morphology and favorable interface temperature conditions between adjacent layers. This demonstrated that a PET recycling process that was tolerant of HDPE contamination and avoided sophisticated compatibilization schemes was possible. With the processing limitations requiring adapted printing strategies, such a low-cost process enabled the printing of quality parts with accuracies comparable to those which can be obtained with high purity, commercially recycled filaments. In future work, both practical and fundamental issues should be considered. Firstly, the recycling logistics, which include waste collection and sorting, must be addressed to ensure that the contamination level remains acceptable so that adequate 3D printing applications can be identified. Secondly, a more systematic investigation of the influence of the process conditions should be conducted. The focus should be on the speed of printing, the resulting shape of the HDPE nodules, and the interface temperature. This information would enable us to better understand the key process influences on the mechanical performance of complex 3D-printed parts.

## Figures and Tables

**Table 1:** Characteristics of the extruded filaments.

Nomenclature	PET Fraction [wt%]	HDPE Fraction [wt%]	Color	Diameter [mm]
E-PET0	100%	0%	Transparent	1.72 ± 0.03
E-PET2	98%	2%	Grey	1.75 ± 0.04
E-PET5	95%	5%	Yellow	1.76 ± 0.03
E-PET10	90%	10%	Orange	1.71 ± 0.06

**Table 2:** Summary of preparations and tests.

Name	Composition	TGA	DSC	Tensile Tests	SEM
PET	Flakes of recycled PET	x			
HDPE	Flakes of recycled HDPE	x			
E-PET0	Extruded recycled PET		x		x
E-PET2	Extruded recycled PET with 2 wt% recycled HDPE		x		x
E-PET5	Extruded recycled PET with 5 wt% recycled HDPE		x		x
E-PET10	Extruded recycled PET with 10 wt% recycled HDPE		x		x
P-PET0	Printed recycled PET		x	x	x
P-PET2	Printed recycled PET with 2 wt% recycled HDPE		x	x	x
P-PET5	Printed recycled PET with 5 wt% recycled HDPE		x	x	x
P-PET10	Printed recycled PET with 10 wt% recycled HDPE		x	x	x
I-PET0	Injected extruded recycled PET (after extrusion)		x	x	x
I-PET5	Injected extruded recycled PET with 5 wt% recycled HDPE		x	x	x
PET_C	Commercial virgin PET filament		x		
PET_CR	Commercial recycled PET filament		x		
P-PET_C	Printed commercial virgin PET			x	
P-PET_CR	Printed commercial recycled PET			x	

**Table 3:** Young’s modulus and maximum tensile stress for printed and injection-molded HDPE-PET blends.

Name	Single Prints		Three Prints	
	Young’s Modulus [GPa]	Max Stress [MPa]	Young’s Modulus [GPa]	Max Stress [MPa]
P-PET0	1.7 ± 0.3	44.8 ± 7.7	2.1 ± 0.1	47.9 ± 3.2
P-PET2	2.1 ± 0.1	51.7 ± 1.1	2.2 ± 0.1	48.5 ± 0.9
P-PET5	2.1 ± 0.0	49.5 ± 1.7	1.8 ± 0.2	37.1 ± 2.6
P-PET10	2.1 ± 0.2	45.6 ± 1.8	2.1 ± 0.1	34.4 ± 3.3
P-PET_C	2.7 ± 0.1	65.1 ± 2.4	2.7 ± 0.1	59.5 ± 2.1
P-PET_CR	2.0 ± 0.0	32.3 ± 13.7	1.9 ± 0.1	48.8 ± 2.3
I-PET0	2.3 ± 0.5	44.4 ± 4.2		
I-PET5	2.9 ± 0.7	34.2 ± 1.6		

**Table 4:** Glass transition Tg, and melting point Tm, of HDPE and PET and crystallinity of PET in blends. Single prints are indicated as ‘1×’ and three prints as ‘3×’, respectively.

Name	Tg PET (°C)	Tm HDPE (°C)	Tm PET (°C)	Xc PET	
				1st Heating	Cooling
E-PET0	73.4	-	253.1	19%	27%
E-PET2	75.6	128.3	252.3	20%	27%
E-PET5	73.6	128.1	251.7	25%	25%
E-PET10	74.0	129.1	252.2	23%	26%
I-PET0	74.2	-	255.8	23%	35%
I-PET5	75.1	129.4	256.4	16%	32%
P-PET0 (1×)	76.4	-	251.6	13%	25%
P-PET2 (1×)	74.8	*No signal*	252.3	19%	25%
P-PET5 (1×)	74.5	128.6	252.4	23%	26%
P-PET10 (1×)	73.9	128.5	252.3	22%	27%
P-PET0 (3×)	75.7	-	252.8	18%	29%
P-PET2 (3×)	73.9	*No signal*	252.8	18%	27%
P-PET5 (3×)	76.3	128.1	252.7	21%	26%
P-PET10 (3×)	75.9	129.3	253.7	22%	27%
PET_C	76.1	-	-	0%	0%
PET_CR	78.1	-	-	0%	0%
P-PET_C (1×)	68.4	-	-	0%	0%
P-PET_CR (1×)	79.2	-	-	0%	0%
P-PET_C (3×)	68.1	-	-	0%	0%
P-PET_CR (3×)	78.4	-	-	0%	0%

## Data Availability

All experimental data are available upon reasonable request to the corresponding author.
